# Psychometric properties of the Chinese version of the Trunk Impairment Scale in people with a stroke

**DOI:** 10.1186/s12955-021-01730-y

**Published:** 2021-03-10

**Authors:** Jie Zhao, Janita Pak Chun Chau, Yuli Zang, Kai Chow Choi, Rong He, Yali Zhao, Xiaoqi Xiang, Qin Li, David R. Thompson

**Affiliations:** 1 The Nethersole School of Nursing, Faculty of Medicine, The Chinese University of Hong Kong, Room 825,8/F, Esther Lee Building, Shatin, New Territories Hong Kong; 2 School of Nursing, Yunnan University of Traditional Chinese Medicine, Kunming, Yunnan China; 3 Yunnan Provincial Hospital of Traditional Chinese Medicine, Kunming, Yunnan China; 4 Kunming Municipal Hospital of Traditional Chinese Medicine, Kunming, Yunnan China; 5 School of Nursing and Midwifery, Queen’s University Belfast, Belfast, UK

**Keywords:** Trunk Impairment Scale, Sitting balance, Stroke, Postural balance, Psychometrics, Reliability, Validity

## Abstract

**Background:**

The Trunk Impairment Scale (TIS) has been translated into Chinese, but the psychometric properties of the Chinese version of the TIS (TIS-C) have not yet been established. We aimed to examine the reliability and validity of the TIS-C for assessing sitting balance among Chinese people with a stroke.

**Methods:**

A descriptive, cross-sectional design was used. We recruited a convenience sample of 170 subacute stroke patients aged 18 years or over from the neurology departments of four traditional Chinese medicine hospitals in China. Patients completed the TIS-C, the Berg Balance Scale and the Modified Barthel Index. The psychometric properties of the TIS-C were examined to establish test–retest reliability, internal consistency, equivalence, and content, criterion, and construct validity.

**Results:**

Intraclass correlation coefficients for inter-rater and intra-rater reliability ranged from 0.75 to 0.89 and from 0.90 to 0.97, respectively. The TIS-C Cronbach α was 0.86. The strong correlation between the total score of the TIS-C and the Berg Balance Scale (*r*_*s*_ = 0.81, *p* < 0.001) or Modified Barthel Index (*r*_*s*_ = 0.84, *p* < 0.001) suggested good concurrent and convergent validity, respectively. Known-group validity was supported by the significant difference (*p* < 0.001) in TIS-C scores between participants with mild and moderate stroke.

**Conclusions:**

The TIS-C is a valid and reliable tool for assessing static and dynamic sitting balance as well as coordination of trunk movement among stroke survivors with mild and moderate stroke.

## Background

Stroke is the leading cause of death and disability worldwide [1]. In China, stroke is the major cause of disability which, though variable, includes structural or functional impairments, activity limitations, and social participation restrictions [2]. The persistent motor and sensory deficits commonly found in people with a stroke are closely associated with impairments in balance [3].

One aspect of such impairments is sitting balance, which is necessary for the performance of functional activities such as dressing and eating in a sitting position. Indeed, sitting balance is an important indicator of mobility and functional outcomes following a stroke [4–6]. Sitting balance assessment measures are therefore necessary to determine the impact of a stroke on a survivor’s life and also the efficacy of treatment and rehabilitation. A variety of instruments, such as the Function in Sitting Test [7], the Modified Functional Reach Test [8], and the Sit-and-Reach Test [9], may be used to measure sitting balance of stroke survivors. A systematic review of the psychometric properties of 14 clinical sitting measurement scales (in 39 papers) found that the methodological quality of the aforementioned tools was rated as poor or fair, whereas the Trunk Impairment Scale (TIS) demonstrated the most promising performance in psychometric properties [10].

The TIS was designed to measure motor impairment of the trunk after stroke: it assesses static and dynamic sitting balance and trunk coordination in a sitting position [11]. It consists of three subscales: static sitting balance, dynamic sitting balance, and coordination. The original English version of the TIS [11] and the Italian [12], Korean [13] and Turkish [14] versions reported it to have satisfactory reliability and validity. Although the Chinese version of TIS (TIS-C) was available [15], its psychometric properties have not yet been established. The aim of this study was to examine the reliability and validity of the TIS-C among Chinese subacute stroke survivors.

## Methods

### Phase 1

Approval to evaluate the psychometric properties of the TIS was obtained from the authors of the original version. A bilingual translator, a PhD student specializing in stroke but without prior knowledge of the original version, back translated the existing TIS-C into English. The back translated version was compared with the original version by an expert panel comprising three PhD students. The panel indicated that no revision was needed. The content validity of the TIS-C was then established by an expert panel comprising one physician, two physiotherapists, and two registered nurses specializing in stroke rehabilitation. The panel used the 4-point Likert scale (1 = ‘not relevant’ to 4 = ‘very relevant’) to evaluate the relevance of each TIS-C item to sitting balance [16]. The item- and scale-level content validity (I-CVI and S-CVI, respectively) were calculated according to established methods [17]. I-CVI is the percentage of individual items of the scale (TIS-C) with a score of over 2 points, while S-CVI is the mean of all I-CVIs for each item respectively. The I-CVI and S-CVI of the TIS-C was 1.0, indicating perfect relevancy to sitting balance or excellent content validity at both item and scale level.

### Phase 2

#### Setting

This study was conducted during July to September 2019 in four neurology departments of traditional Chinese medicine (TCM) hospitals in Kunming, China. The hospitals were selected by convenience due to resource limitations.

#### Participants

Based on guidelines for the translation, adaptation and validation of instruments for cross-cultural use, a sample size of 170 participants was required to determine the psychometric properties of the TIS-C (10 participants per item; 17 items) [18]. Thus, a convenience sample of 170 stroke survivors was recruited from the four hospitals. Inclusion criteria were: (1) age ≥ 18 years; (2) clinical diagnosis of an ischemic or hemorrhagic stroke; (3) in the subacute phase of stroke (two weeks to six months after stroke) [19]; (4) within 72 h after admission to the neurology unit; (5) able to sit independently with or without cushions; and (6) able to communicate and provide informed consent in Chinese. Exclusion criteria were: (1) unstable vital signs; (2) impaired cognitive functions (Abbreviated Mental Test ≤ 7) [20]; (3) severe hearing or visual impairment; (4) severe complications after stroke (e.g., compromised cognitive ability, receptive aphasia, and venous thrombosis); or (5) National Institutes of Health Stroke Scale (NIHSS) score ≥ 16 [21].

### Instruments

#### Chinese version of the Trunk Impairment Scale

The 17-item TIS-C comprises three subscales: static sitting balance (3 items), dynamic sitting balance (10 items), and coordination (4 items) [11]. Each item is scored on a 2-, 3- or 4-point ordinal scale from 0 to 3, with the maximal scores for the static and dynamic sitting balance and coordination subscales that can be attained being 7, 10 and 6. The total scores for the TIS-C range from 0 to 23, representing the lowest to the highest level of body balancing function [11].

#### Chinese version of the Berg Balance Scale

The 14-item Chinese version of the Berg Balance Scale (BBS-C) is a widely used balance assessment tool in China [22]. Each item is scored from 0 to 4, with a total score range from 0 to 56. A higher score indicates better balance control [23]. The reliability and validity of the BBS-C has been established in stroke survivors, with intra and inter-rater intraclass correlation coefficients (ICCs) of 0.97 – 0.99 and 0.99, respectively [24].

Participants’ demographic and clinical data, including gender, age, education, occupation, activities of daily living (ADL) measured by the Modified Barthel Index (MBI) (range 0–100) [25], stroke type, duration of stroke, involved side, and severity of stroke graded by NIHSS (range 0–42), were recorded. NIHSS scores of 0–6 indicate mild stroke, 7–16 moderate stroke, and > 16 severe stroke [21].

### Examining the psychometric properties of the TIS-C

Equivalence (inter-rater reliability), test–retest reliability (intra-rater reliability), internal consistency, and content, criterion (concurrent), and construct (convergent and known-group) validity of the TIS-C were determined [16, 26].

Two assessors (A: principal investigator; B: research assistant), both registered nurses, examined inter-rater reliability over six weeks. As recommended for test–retest reliability [27], 50 participants were randomly selected to participate in the second evaluation two or three days after the initial assessment.

The BBS-C and MBI were also administered to determine concurrent and convergent validity. Known-group validity was assessed by examining the difference between the TIS-C mean scores of participants with mild stroke and those with moderate stroke (there should be significant differences in the function of sitting balance between the two groups). We hypothesized that an improvement in sitting balance would imply an improvement in ADL. In addition, participants with moderate stroke were expected to receive lower TIS-C scores than those with mild stroke.

### Data collection

The two assessors, bilingual and experienced in clinical neurological assessment, trained themselves in the use of the TIS by viewing the video produced by the original scale developer [28]. Assessor A approached eligible stroke survivors and explained the study to them. Both assessors followed the same instructive protocol to guide participants to take the test, and simultaneously scored participants’ performances independently. All participants had three opportunities to try the required movements, and the highest score obtained was used for further analysis [11]. Only assessor A carried out the second assessment within three days to determine intra-rater reliability. To minimize recall bias, assessor A filled out the score sheet without calculating the total score of the scale and its subscales until all participants had completed the two assessments.

The BBS-C and MBI were administered to participants by assessor A immediately after the TIS-C assessment. Demographic and clinical data were retrieved from the medical records of participants or by direct requests to them or their family.

### Ethical considerations

The authors obtained permission to use the TIS and were granted approval from the host institutions to conduct the study. Informed written consent was obtained from participants before data collection. All data were kept anonymous, secure and strictly confidential and used for research purposes only.

### Statistical analysis

Statistical analyses were conducted using the SPSS version 25.0. Descriptive statistics were used to summarize sociodemographic and clinical. The normality of continuous data was checked by skewness statistic and normal probability plot, which showed that the NIHSS, TIS-C, BBS-C, and MBI scores were non-normally distributed. Thus, nonparametric analyses (e.g., Spearman rank correlation analysis and Mann–Whitney U test) were conducted.

The S-CVI and I-CVI were calculated according to the suggested equation and a CVI of at least 0.80 was considered acceptable for content validity [17]. Inter- and intra-rater reliability for total and subscale scores of TIS-C were determined by ICCs, with a cutoff of > 0.75 indicating good reliability and an ICC of 0.5–0.75 suggesting acceptable reliability [29]. Kappa and weighted kappa values were calculated for dichotomous and ordinal variables as measured by scale items, respectively. The cutoff kappa and weighted kappa, and their corresponding degrees of agreement range from no agreement (≤ 0), none to slight (0–0.20), fair (0.21–0.40), moderate (0.41–0.60), substantial (0.61–0.80), and almost perfect agreement (0.81–1.00) [30]. Internal reliability analysis was performed to examine internal consistency, with a Cronbach’s α of > 0.70 suggesting acceptable internal reliability [31]. Spearman rank correlation analysis was employed to calculate the correlation coefficient between the scores of TIS-C, and BBS-C or MBI. A correlation coefficient above 0.7 indicates a high correlation [31]. Mann–Whitney U test was used to compare the distribution of TIS-C scores between those with mild stroke and moderate stroke. The level of significance was set at 0.05 for 2-sided tests for all analyses.

## Results

### Sample characteristics

The Chinese subacute stroke survivors (n = 170) had a mean age of 62.7 ± 9.4 (range 22 to 82) years. Sixty-one percent (n = 103) were male, and 81% (n = 137) had suffered an ischemic stroke. The average duration of stroke was 57.4 ± 49.3 days, with a NIHSS median score of 2 and interquartile range of 4 (Table 1).

**Table 1:** Participants’ sociodemographic & clinical characteristics (n = 170)

Group/subgroup	N	%
Gender		
Male	103	60.6
Female	67	39.4
Education level		
Primary	26	15.3
Secondary	73	42.9
Tertiary	71	41.8
Without degree	55	32.4
Bachelor/master degree	16	9.4
Stroke type		
Ischemic	137	80.6
Hemorrhagic	33	19.4
Impaired body side		
Left	87	51.2
Right	83	48.8
Severity category by NIHSS		
Mild	54	31.8
Moderate	116	68.2

NIHSS: National institutes of health stroke scale

Fifty of the 170 participants were involved in the second assessment. No significant differences in sociodemographic and clinical characteristics were found between these 50 and the other 120 participants (*p* > 0.05).

### Reliability

The ICCs of TIS-C scores as measured by two assessors for the same participant were 0.75 for static sitting balance, 0.94 for dynamic sitting balance, 0.89 for coordination, and 0.96 for total TIS-C score, suggesting moderate to high inter-rater reliability. The ICCs of TIS-C scores as measured at two measure points were 0.90 for static sitting balance, 0.93 for dynamic sitting balance, 0.90 for coordination, and 0.97 for total TIS-C score, suggesting high test–retest reliability (Table 2).

**Table 2:** ICC for inter-rater and intra-rater reliability

Scale/dimension	Inter-rater reliability (N = 170)		Intra-rater reliability (N = 50)	
	ICC	95% CI	ICC	95% CI
Total score	0.96	(0.95, 0.97)	0.97	(0.95, 0.98)
Static sitting balance	0.75	(0.67, 0.81)	0.90	(0.83, 0.94)
Dynamic sitting balance	0.94	(0.92, 0.95)	0.93	(0.87, 0.96)
Co-ordination	0.89	(0.86, 0.92)	0.90	(0.84, 0.94)

ICC: Intraclass correlation coefficient; CI: confidence interval

The kappa and weighted kappa of TIS-C scores ranged from moderate (0.41) to almost perfect agreement (0.89) for inter-rater agreement, and ranged from substantial (0.63) to almost perfect agreement (0.91) for intra-rater agreement (Table 3). The Cronbach α for the total TIS-C was 0.86, 0.83 for the dynamic sitting balance subscale, and 0.92 for the coordination subscale. The results implied good internal reliability of the TIS-C (Table 4).

**Table 3:** Inter-rater and intra-rater agreement statistics

Dimension/item	Inter-rater agreement (N = 170)		Intra-rater agreement (N = 50)	
	Kappa	%^a^	Kappa	%^a^
Static sitting balance				
Item 1	1^b^	100	1^b^	100
Item 2	1^b^	100	1^b^	100
Item 3	0.63^c^	79.41	0.80^c^	88.00
Dynamic sitting balance				
Item 4	0.87	93.54	0.71	93.00
Item 5	0.81	92.35	0.77	94.00
Item 6	0.89	94.71	0.79	90.00
Item 7	0.88	90.25	0.82	92.00
Item 8	0.71	92.94	0.85	98.00
Item 9	0.84	93.53	0.74	94.00
Item 10	0.41	85.88	0.81	96.00
Item 11	0.63	84.12	0.83	94.00
Item 12	0.45	90.59	1^b^	100
Item 13	0.55	81.76	0.63	88.00
Co-ordination				
Item 14	0.83^a^	92.94	0.70^a^	88.00
Item 15	0.84	92.94	0.77	90.00
Item 16	0.81^a^	90.59	0.91^a^	96.00
Item 17	0.84	92.35	0.84	92.00

^a^Percentage of inter- or intra-rater agreement;

^b^Participants obtained the same score given their body function at mild or moderate severity of impairment instead of deteriorating status

^c^Weighted kappa

**Table 4:** Internal consistency of Trunk Impairment Scale (n = 170)

Scale/dimension	No. of items	Cronbach’s α
Total scale	17	0.86
Static sitting balance	3	Not applicable^a^
Dynamic sitting balance	10	0.83
Co-ordination	4	0.92

^a^None of participants had sever static balancing impairment that a constant score (2) was assigned for all

### Criterion and construct validity

Strong correlations were found between the TIS-C score, and that of the BBS-C (*r*_*s*_ = 0.81, *p* < 0.001) or MBI (*r*_*s*_ = 0.84, *p* < 0.001), suggesting concurrent and convergent validity. Significant difference between the TIS-C score distribution of participants with mild stroke (n = 54, median 22, interquartile range 4) and moderate stroke (n = 116, median 14, interquartile range 4) was detected, supporting the known-group validity of the TIS-C (Z = 9.79, *p* < 0.001).

## Discussion

This study demonstrates that the TIS-C is a valid and reliable measure for use among Chinese people with a stroke. We provide evidence that the TIS-C has satisfactory intra- and inter-rater reliability, internal consistency, and concurrent and construct validity among stroke survivors. Overall, the TIS-C demonstrates similar psychometric properties to Belgian [11], Italian [12], Korean [13], and Turkish [14] versions of the instrument.

While none of the previous studies reported the CVI of the TIS, the CVI of the TIS-C in our study was 1.0, suggesting that it can adequately measure intended sitting balance for stroke survivors [17]. However, modified versions of the TIS have been developed, such as the static sitting balance subscale being removed in the second iteration (2.0) of the TIS because it did not fit the Rasch model [32, 33], and the 6-item TIS Norwegian version being reconstructed based on Item Response Theory [34]. We did not adopt the TIS 2.0 as we believe that the TIS often works better as a whole to fully capture the degree of stroke survivors’ sitting balance control. In addition, we intended to examine whether the static sitting balance subscale was appropriate for use among a wider range of stroke conditions. These considerations may guide specific rehabilitation interventions, such as choosing optimal posture, and prognostic estimations. Future studies could compare the performance of the Chinese version of TIS 2.0 and TIS-C using factor or Rasch analysis.

The whole TIS-C and its subscales have shown excellent reliability. For the whole scale, all percentages of agreement between raters exceeded 81%. The inter-rater reliability of each item ranged from 0.41 to 0.89. This variability has also been reported in previous studies and may be a result of a small number of raters [11, 12]. The TIS-C demonstrates satisfactory internal consistency, similar to findings from other language versions [11, 12, 14]. Compared with a previous study [12], a major difference in our study relates to the static sitting balance subscale. As we recruited mild to moderate stroke survivors, the score of a specific item ‘Patient can maintain starting position for 10 s’ was therefore constant (i.e., 2 points). Consequently, it was not appropriate to calculate its Cronbach’s α. While Lombardi and Paci [12] reported a Cronbach’s α 0.83 for the static sitting balance subscale among Italian subacute stroke survivors, they did not report their stroke severity, though, as indicated by the MBI (39.6 ± 15.4), the level of ADL among their participants was lower than ours.

In China, post-stroke balance, including sitting balance, is measured largely by the BBS [22], the balance subscale of the Fugl-Meyer Assessment (FMA) [35], and computerized body balance devices, such as Smart-EquiTest [32]. The BBS is not a stroke-specific sitting balance measure [23], while the FMA balance subscale fails to capture dynamic sitting balance [35]. Further, computerized body balance devices are uncommon, expensive and time- or energy-consuming, which limit their usability [22, 36]. A stroke-specific, easy-to-use tool with satisfactory psychometric properties for rapid assessment of sitting balance is therefore desirable.

Our study provides convincing evidence about the appeal of using the TIS-C. It is quick to score, taking 2 to 18 min [11]. Nurses and peers could use it to assess and communicate a stroke survivor’s condition. Besides, the TIS has been validated among patients with other conditions such as neuromuscular diseases [37], traumatic brain injury [38] and Parkinson’s disease [39]. Further research might examine the TIS-C in conditions in which sitting balance is impaired.

A limitation of the TIS is that it only identifies impairment of sitting balance and deficiencies at the body function and structure level of the International Classification of Functioning, Disability and Health (ICF) [10, 40]. It lacks elements to evaluate sitting balance limitation or restriction at the activities and participation level of the ICF. Therefore, it would be beneficial to add other related instruments if it is necessary to measure sitting balance in social participation. Another limitation of this study is that we only recruited mild to moderate subacute stroke survivors and in relatively small numbers in TCM hospitals. Thus, our sample and findings may not be representative of or applicable to all subacute stroke survivors in China. Therefore, further investigation regarding the applicability of theTIS-C among stroke survivors with severe impairment and in other settings is warranted.

## Conclusions

The TIS-C is a reliable and valid tool to monitor sitting balance among Chinese people with a mild to moderate subacute stroke. Though the original TIS measures sitting balance among stroke survivors with high reliability and validity, the psychometric properties of the TIS-C have not been previously assessed. As recognized, the routine use of the TIS-C in assessing sitting balance at the early phase of mild to moderate physical impairment is conductive to the monitoring of dysfunctional status towards optimal recovery among stroke survivors in usual practice. Further studies to examine the use of the TIS-C among other stroke survivor populations and setting are warranted.

## Data Availability

The datasets used and/or analyzed during the current study are available from the corresponding author on reasonable request.

## Abbreviations

TIS: Trunk Impairment Scale
TIS-C: The Chinese version of the Trunk Impairment Scale
RNs: Registered nurses
CVI: Content validity
I-CVI: Item-level content validity
S-CVI: Scale-level content validity
TCM: Traditional Chinese medicine
NIHSS: National Institutes of Health Stroke Scale
BBS-C: Chinese version of Berg Balance Scale
ICCs: Inter-rater intraclass correlation coefficients
ADL: Activities of daily living
MBI: Modified Barthel Index
FMA: Fugl–Meyer Assessment
ICF: International Classification of functioning, Disability and Health
